# ACSL4 inhibition prevents macrophage ferroptosis and alleviates fibrosis in bleomycin-induced systemic sclerosis model

**DOI:** 10.1186/s13075-023-03190-9

**Published:** 2023-10-26

**Authors:** Dianyu Cao, Jina Zheng, Zheng Li, Yong Yu, Zengrui Chen, Qiang Wang

**Affiliations:** 1 Department of Dermatology, Zhongshan Hospital, Fudan University, No.180 Fenglin Road, Xuhui District, Shanghai, 200032 P.R. China; 2 Laboratory Animal Division, Institute of Clinical Science, Zhongshan Hospital, Fudan University, 180 Fenglin Road, Shanghai, 200032 P.R. China; 3 Key Laboratory of Viral Heart Diseases, Ministry of Public Health, Zhongshan Hospital, Fudan University, Shanghai, 200032 P.R. China; 4 Department of Intensive Care Medicine, Yuhuan People’s Hospital, No. 18 Changle Road, Yucheng Street, Yuhuan City, Zhejiang 317600 P.R. China

**Keywords:** Ferroptosis, ACSL4, Macrophage, Fibrosis, Calpain, Systemic sclerosis

## Abstract

**Background:**

Systemic sclerosis (SSc), with unclear pathophysiology, is a paradigmatic rheumatic disease of immunity dysfunction-driven multi-organ inflammation and ultimate fibrosis. Pathogenesis breakthroughs are urgently needed for available treatments halting its unremitting stiffness. This study aims to investigate whether ferroptosis can regulate the progressive SSc fibrosis.

**Methods:**

In vivo, bleomycin (BLM)-induced mice model was subjected to ferroptosis detection using western blotting, malondialdehyde (MDA), and glutathione (GSH) assays. Pharmacological inhibitor of the acyl-CoA synthetase long-chain family member 4 (ACSL4) was utilized to explore its potential therapeutic effects for fibrosis, from histological, biochemical, and molecular analyses. In vitro, bone marrow-derived macrophages (BMDM) were activated into inflammatory phenotype and then the relationship was evaluated between activation level and ferroptosis sensitivity in lipopolysaccharide (LPS) incubation with gradient concentrations. The potential calpain/ACSL4 axis was analyzed after calpain knockdown or over-expression in Raw264.7.

**Results:**

Both skin and lung tissue ferroptosis were present in SSc mice with enhanced ACSL4 expression, while ACSL4 inhibition effectively halted fibrosis progressing and provides protection from inflammatory milieu. Meanwhile, a positive regulation relationship between LPS-induced macrophage activity and ferroptosis sensitivity can be observed. After calpain knockdown, both inflammatory macrophage ferroptosis sensitivity and ACSL4 expression decreased, while its over-expression renders ACSL4-envoking condition. Also, calpain pharmacological inhibition reduced both ferroptosis and fibrosis aptitude in mice.

**Conclusions:**

ACSL4 induces inflammatory macrophage ferroptosis to aggravate fibrosis progressing. ACSL4 and its upregulators of calpains may be potential therapeutic targets for BLM model of SSc.

**Supplementary Information:**

The online version contains supplementary material available at 10.1186/s13075-023-03190-9.

## Introduction

Systemic sclerosis (SSc) is a chronic rheumatic disease characterized by immunity dysfunction, inflammation, fibrosis, vasculopathy of the skin, and internal organ [1–4]. Unclear SSc pathophysiology lies in the altered balance of immune system, as well as the interplay between unremitting inflammation and advancement of fibrosis [4]. Frequently associated with skin stiffness, severe pulmonary involvement cannot be overlooked. Among the patients of systemic sclerosis, interstitial lung disease (ILD) is the leading cause of death, with prevalence up to 30% and 10-year mortality up to 40% [2]. Although remarkable breakthroughs have been made in the management of organ complications, a curative treatment is urgently needed to prevent the progressing fibrosis both in skin and inner organs [3, 4]. Recent bioinformatics research at the single-cell transcriptome level unveils ferroptosis has been detected in SSc-ILD and proinflammatory factors can drive changes in ferroptosis [5]. However, the precise molecular mechanisms underpinning these responses remain to be elucidated, and no cure is available to correct this life-threatening condition.

Ferroptosis, as a newly discovered programmed cell death (PCD), is recently wildly researched in autoimmune disorders and rheumatic diseases, especially with a recent study suggesting its interplay in systemic autoimmunity of systemic lupus erythematosus (SLE) [6]. For ferroptosis detection, it is regulated by several wildly accepted factors: pivotal proteins expression, cellular redox homeostasis disturbance and membrane phospholipid peroxidation, metabolic iron input and Fenton reaction [7–10]. Pivotal markers such as acyl-CoA synthetase long-chain family member 4 (ACSL4) are known for their significant regulatory effects on ferroptosis in many immunity disorders [6, 8–11]. ACSL4 drives ferroptosis based on its ability to ligate long-chain polyunsaturated fatty acids (PUFAs), which are one of the main targets of lipid peroxidation [7–9]. Therefore, the production of PUFAs mediated by ACSL4 leads to increased sensitivity to ferroptosis [9]. Previous studies showed that genetic loss of *ACSL4* or its pharmacological inhibition is a principal mechanism in desensitizing cells to ferroptosis [12]. Persistent monocyte-derived macrophage (Mφ) accumulation leads to kinase (ERK)-dependent 5-lipoxygenase (ALOX5) over-expression and accumulation of its metabolite leukotriene B4 (LTB4), which triggers expression of ACSL4 a ferroptosis promoting gene in lung epithelial cells [13]. Monocyte/macrophage is the critical cellular component of innate immunity. It is apparent that inflammatory macrophage, or also called M1, correlates with fibrotic initiation and expedites fibrosis progression in SSc [4, 14, 15]. Meanwhile, study showed a lipopolysaccharide (LPS)-trained macrophage present biphasic effect on inflammation-induced fibrosis like SSc [15]. Under a SSc context, the inflammatory macrophages secrete cytokines and mediators like inducible nitric oxide synthase (iNOS), reactive oxygen species (ROS), interleukin 1 beta (IL-1β), and interleukin 6 (IL-6), thus establishing a microenvironment supportive of fibroblasts proliferation and excessive collagen accumulation [2, 4]. Also, secreted chemokines themselves also recruit more inflammatory cells, leading to a positive feedback loop [4]. It is important to stop the overshooting inflammatory response for fibrosis early intervention [4]. It is found that some of the secreted inflammatory mediators like iNOS and ROS also have effects on lipid peroxidation, iron homeostasis, and amino acid metabolisms in various disease models, which can influence the macrophage susceptibility to ferroptosis [10, 16]. Previous studies have showed that in brain, monocytes, and polarized macrophages (M1 or M2) indicate a different sensitivity to ferroptosis [17]. However, the mechanisms underlying of macrophage ferroptosis in SSc remain to be defined.

Calpains belong to a family of calcium-dependent, cysteine proteases associating with many cell signaling pathway, including apoptosis [18]. The family has two most extensive studied heterodimer members, calpain 1 or calpain 2, and the large catalytic subunit of which are encoded by the *CAPN1* and *CAPN2* respectively while the shared small subunit is encoded by *CAPNS1* [18]. Our recent study showed that knockout *CAPNS1* in myeloid cell can prevent its polarization reducing the inflammation and fibrosis in lung tissues of SSc [19]. Interestingly, a latest study showed calpain has a mediating effect on ferroptosis in the radiation-induced lung injury [20]. However, the role and mechanisms of calpain as a regulating bridge from polarized macrophage ferroptosis to SSc fibrosis and inflammation remain not fully understood.

In this study, we hypothesized that ferroptosis is present with increased ACSL4 mainly regulating macrophage sensitivity, leading to fibrosis and inflammation in BLM model of SSc. Meanwhile, calpains may work as the upstream regulator of ACSL4 expression, which can prevent macrophage increased sensitivity to ferroptosis, thereby reducing the disease progression of BLM model mice.

## Materials and methods

### Drugs and chemicals

All the drugs and chemicals used in this study are listed as follows (Table 1).

**Table 1:** Drugs and chemicals

Chemicals	Vendor	Catalog No.	City, Country
LPS	Sigma-Aldrich	L4391	USA
Erastin	Abmole	M2679	Huston, USA
Macrophage colony-stimulating factor (M-CSF)	MCE Chemical	HY-P7085	New York City, USA
PD150606	MCE Chemical	HY-100529	New York City, USA
Rosiglitazone (ROSI)	MCE Chemical	HY-17386	New York City, USA
Bleomycin (BLM)	Nippon Kayaku	/	Tokyo, Japan

### Animal model

All animal procedures were approved by the Institutional Animal Care and Use committee of Zhongshan Hospital, Fudan University (2022-042) in compliance with the guidelines for the Care and Use of Laboratory Animals published by the National Academy Press (NIH Publication No.85-23, revised 1996). Balb/c mice (female, 4–6 weeks old, 18–22g) were purchased from Shanghai JieSiJie Laboratory Animals Co., LTD. All these mice were housed in the animal facility of Zhongshan Hospital, Shanghai, with a 12-h dark/light cycle. Bleomycin powder was dissolved in sterilized PBS at a concentration of 1.0 mg/ml, filtrated through a 0.22-μm filter (Milipore, USA) and then stored at 4 ℃. Mice were subcutaneously injected with 0.1ml BLM on the lower back at exact location daily for 4 weeks. The moderate disease group injected with a concentration of 0.5 mg/ml, while the severe group with 1.0 mg/ml.

### Animal treatment and groups

All the mice were randomly assigned into the following groups of 8 animals each with comparable mean body weight:① Blank control: received PBS; ② BLM group: received BLM; ③ ACSL4 inhibition: Balb/c mice with daily subcutaneously injection of BLM, then treated with intraperitoneal injection of ROSI (daily, 0.5 mg/kg, 0.1 ml/day dissolved in sterilized PBS, lasts for 21 days); ④ Calpain inhibition: Balb/c mice with daily subcutaneously injection of BLM, then treated with intraperitoneal injection of PD150606 (daily, 3 mg/kg, 0.1 ml/day dissolved in DMSO and PBS, lasts for 21 days). After treatment, all mice were sacrificed to collect skin tissue, pulmonary tissue, and serum.

### Histological analysis

Left lung tissues and local skin tissues were inflated and immersed in fresh 4% neutral buffered paraformaldehyde for 36 h at room temperature, then embedded in paraffin.

Four-micrometer-thick sections were stained with hematoxylin and eosin (H&E), and we randomly selected 5 high-power field (HPF, ×200) of each slide. Semi-quantitative measurement of inflammation in lung tissue was graded into the following categories [21] and two independent researchers are involved in grading the slices: grade 0, normal lung tissue without inflammation, scored 0; grade 1, minimal alveolitis (+), widened alveolar septa due to inflammatory cells infiltration, the lesions confirmed to less than 20% of the whole lung, scored 1.0; grade 2, moderate alveolitis (++), the lesions extended to 20% to 50% of the whole lung, scored 2.0; grade 3, severe alveolitis (+++), diffuse lesions in more than 50% of the whole lung, scored 3.0.

Likewise, sections of skin and lung tissues were stained with Masson’s trichrome for fibrosis analysis, calculating the collagen volume fracture (CVF, %) as positive area was stained blue with ImageJ software (NIH, USA).

For skin thickness analysis, we first randomly selected 5 HPF (×200) of each slide for the semi-quantitative measurement. And then, according to each randomly selected field of view, the distance from the epidermis to the subdermis is measured vertically with the ruler function in the K-viewer software.

### Isolation of primary bone marrow-derived macrophages (BMDM)

As the previous study indicated [22], cleaned femurs and tibia bones were cut from the control and BLM disease model mice. Then they were centrifuged to collect bone marrow cells in sterile PBS and plated in Dulbecco’s modified Eagle’s medium (DMEM; Biosharp) additional supplemented with macrophage colony-stimulating factor (M-CSF; 10 ng/ml). After 5 days culture, cells were harvested for later treatment and detection.

### Cell culture and establishment of stable cell lines

Raw264.7 cells were purchased from the American Type Culture Collection (Manassas, VA, USA), and were cultured in DMEM supplemented with 10% heated-inactive fetal bovine serum (FBS; Gibco) and 1% penicillin-streptomycin (Biosharp) at 37 °C with 5% CO_2_.

Sequences of calpain 1 and calpain 2 knockdown and over-expression are listed in Supplementary materials Table 1. Then these sequences were packed in lentivirus for transfection (ZuoRun Biotech, Shanghai, China). After 5 days infection, the infection efficiency was firstly determined by counting the numbers of green fluorescent protein (GFP)-expressing cells under a fluorescence microscope (DMI4000B, Leica Microsystems, Germany).

### Western blotting

Tissues and cells were lysed in a mixture of RIPA lysis buffer (P0013, Beyotime, Shanghai, China) and phenylmethanesulfonyl fluoride (PMSF; ST506, Beyotime, Shanghai, China). Then after total protein collected, the supernatant was quantified by a BCA kit (ST2222, Beyotime, Shanghai, China). Proteins from each sample were subjected to electrophoresis on 12% sodium dodecyl sulfate-poly-acrylamide gel (SDS-PAGE), and then transferred to polyvinylidene fluoride (PVDF) membranes on a semi-dry electro transferring unit (Bio-Rad, USA). PVDF membranes were blocked with 3% Albumin Bovine V for 2 h and incubated with the diluted primary antibodies overnight at 4℃. All the primary antibodies used in this study are listed as follows (Table 2). After incubation, membranes were washed by phosphate-buffered saline with 0.1% Tween-20 (PBST) and incubated with HRP-conjugated secondary antibody (A0208, Beyotime, Shanghai, China) for 2 h. After extensive washing, blots were detected with enhanced chemiluminescence assays.

**Table 2:** Primary antibodies for western blotting

Primary antibody	Vendor	Catalog No.	Working dilution (for western blotting)
ACSL4	Abmart	T510198	1:1000
FTH1	Abmart	T56955	1:1000
GPX4	Abmart	T56959	1:1000
SLC7A11	Abmart	T57046	1:1000
Calpain1	CST	#2556	1:1000
Calpain2	CST	#2539	1:1000
Capns1	Abclonal	A6539	1:1000
Collagen Ia	Abclonal	A5786	1:1000
Collagen III	Abclonal	A3795	1:1000
α-SMA	Abclonal	A17910	1:1000
β-actin	Abclonal	AC026	1:100,000

### RNA extraction and quantitative real-time PCR (qRT-PCR)

Total RNA was extracted using the RNAiso Kit (R0024, Beyotime, Shanghai, China). cDNA was synthesized using the PrimeScript RT Master Mix (RR036A, takara, Janpan), and PCR reaction mixtures were prepared using qPCR SYBR Green Master Mix (CatNo.11202ES08, Yeasen, shanghai, China). The RT-PCR reaction process was as follows: 95 °C for 5 min, followed by amplification in 40 cycles of 95 °C for 10 s, 20 s at 60 °C, and 20 s at 72 °C, then melting in default using the ABI 7900 Real-Time PCR System. The primer sequences are listed as follows. β-Actin served as the internal reference, and the 2−ΔΔCt method was used to quantify the relative expression of mRNA. All the primers used in this study are listed in Supplementary Table 2.

### Cell viability assay

Cell viability was assessed by using the cell counting kit 8 (CCK8; M4839, Abmole) according to the manufacturer’s instructions. Cells were seeded in 96-well plates (20,000 cells per well) and treated with 10 μl erastin or PBS in the incubator for 24 h. Then the culture medium was replaced with 100 μl fresh supplemented DMEM containing 10 μl CCK8 in each well of the plate. The cells were incubated in the cell incubator for 2 h and then the absorbance at 450 nm was measured using the microplate reader.

### Calpain activity assay

The tissue lysates were assay for calpain activity using a calpain Assay Kit (ab65308, Abcam, UK) according to the manufacturer’s instructions. After incubating at 37℃ for 1 h in dark, the calpain activity was detected by the fluorescence counter.

### Hydroxyproline (HYP) measurement

Weighing 100 mg skin and lung tissue for HYP content measurement is followed the manufacturer’s instructions of a HYP Test Kit (A030-2, Jiancheng, Nanjing, China). All sections were examined independently by two investigators in a blinded manner.

### Iron measurements

The fresh blood was collected by heart puncture and immediately centrifuged for the serum. The serum iron level was detected by the Iron Assay Kit (A039-1-1, Jiancheng, Nanjing, China) according to the manufacturer’s instructions.

### Malondialdehyde (MDA) analysis and glutathione (GSH)-oxidized glutathione (GSSG) analysis

The MDA concentration of tissue and cell lysates were assessed using the MDA Assay Kit (S0131, Beyotime, Shanghai, China) according to the manufacturer’s instructions.

Total GSH and GSSG levels of tissue and cell lysates were measured using the GSH-GSSG Assay Kit (S0053, Beyotime, Shanghai, China) according to the manufacturer’s instructions. A standard curve was established by the addition of GSH to PBS, and net GSH concentration was determined as subtract GSSG concentration from total GSH content.

### Immunohistochemical (IHC) analyses

Four-micrometer-thick sections were deparaffinized and dehydrated and the endogenous peroxidase blocked for 15 min. Then after restoring antigen, the sections were incubated with diluted primary antibodies of ACSL4 (1:200), F4/80 (1:200) and secondary antibody EnVision^+^/HRP before visualizing by a DAB kit and evaluating under light microscopy (Nikon, Japan). The number of positive cells (brown) was calculated with Q500IW image analysis system (Leica, Germany) and Image-Pro Plus 6.0 software.

### Statistical analysis

Data were represented as mean ± SD or mean. One-way ANOVA followed by Tukey’s multiple comparison test or Student’s *t* test were applied for calculating statistical differences. The data processing and graphs were performed by using GraphPad Prism software (version: 9.0.0.121) and ImageJ software (version: 1.8.0). *P*-value < 0.05 was considered to be statistically significant.

## Results

### Ferroptosis is present in the BLM-induced SSc mice model

**Fig. 1:**
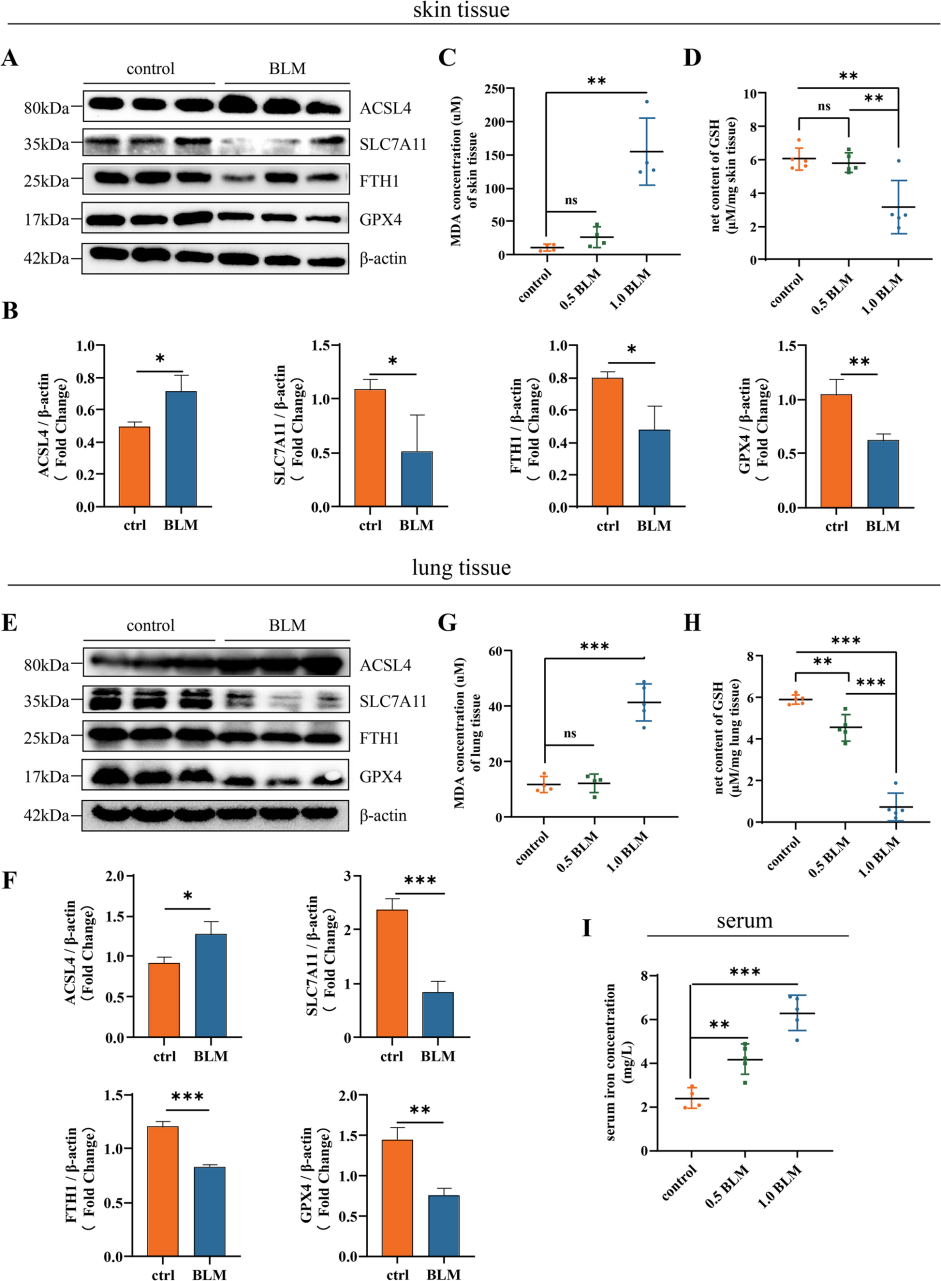
Ferroptosis is present in the BLM-induced SSc mice model. **A**, **B** Western blots for ferroptosis marker of the skin tissue from healthy control mice and BLM-induced mice (1 mg/ml, 0.1 ml/day, for 4 weeks).** C** Relative MDA levels of murine skin tissue in healthy control group, moderate BLM group (0.5 mg/ml BLM, 0.1 ml/day, for 4 weeks), and severe BLM group (1 mg/ml BLM, 0.1 ml/day, for 4 weeks) (*n* = 5). **D** Net GSH concentration of murine skin tissue in above groups (*n* = 5). **E**, **F** Western blots for ferroptosis marker of the lower back lung tissue from healthy control mice and BLM-induced mice (1 mg/ml, 0.1 ml/day, for 4 weeks). **G** Relative MDA levels of murine lung tissue in healthy control group, moderate BLM group, and severe BLM group (*n* = 5). **H** Net GSH concentration of murine lung tissue in above groups (*n* = 5). **I** Serum iron level of each group (*n* = 5). (Data are presented as Mean ± SD *p < 0.05, **p < 0.01, ***p < 0.001)

As a rationale for evaluation of ferroptosis, we first showed that compared to healthy controls, BLM-induced mice models (1.0 mg/ml, 0.1ml, daily) display increased ferroptosis driver ACSL4 protein levels and reductions in ferroptosis suppressors: ferritin heavy chain 1 (FTH1), solute carrier family 7a member 11 (SLC7A11), glutathione peroxidase 4 (GPX4) levels by protein analyses in local induced skin lesions (Fig. 1A, B).

To delineate the ferroptosis-related redox dysregulation changes during fibrotic severity and progression, we first searched literature for the suitable range of BLM concentration injected subcutaneously in Balb/c mice to induce a discernable stiff and rigid local skin. We calculated the final dose that ranges from 0.2 mg/ml (daily, for 4 weeks) to 1.0 mg/ml (daily, for 4 weeks), and 0.5 mg/ml (lasts for 4 weeks) was the most regular BLM regime [18, 23–26]. We selected two concentrations based on the local fibrotic presentation: 0.5 mg/ml daily injection for 4 weeks representing middle dosage and mild fibrosis stage, and 1.0 mg/ml representing advanced dosage and severe fibrosis stage.

We chose malondialdehyde (MDA) as a by-product of lipid peroxidation [27], together with net reduced glutathione (GSH), to address the local skin tissue redox reserve pool. Compared to healthy controls, progressively increased MDA levels (Fig. 1C) and decreased net GSH levels (Fig. 1D) among 0.5 mg/ml group and 1.0 mg/ml group revealed a dynamic downregulation of antioxidant capacity during progression of fibrosis in local skin.

Wilder assessment of the inner organ involvement of ferroptosis presentation was conducted by analyzing pulmonary tissue, for the reason that lung as one of the most frequently affected organs and interstitial lung disease (ILD) as the leading cause of death in SSc [2]. Same tendency of ferroptosis-associated proteins was shown (Fig. 1E, F), in line with elevated MDA (Fig. 1G) and deficiency in net GSH (Fig. 1H), indicating a systemic involvement of ferroptosis in fibrotic tissue. Lastly, significant elevation of circulating iron levels, a key character in ferroptosis, also occurred with BLM injection (Fig. 1I).

Collectively, these results support that wild range of tissue ferroptosis is present in SSc mice and is progressing with BLM concentration injected. Thus, for the rest of mice analyses, we chose a higher concentration group, the 1.0 mg/ml BLM-induced mice models.

### Inhibition of ferroptosis driver ACSL4 attenuates SSc fibrosis

**Fig. 2:**
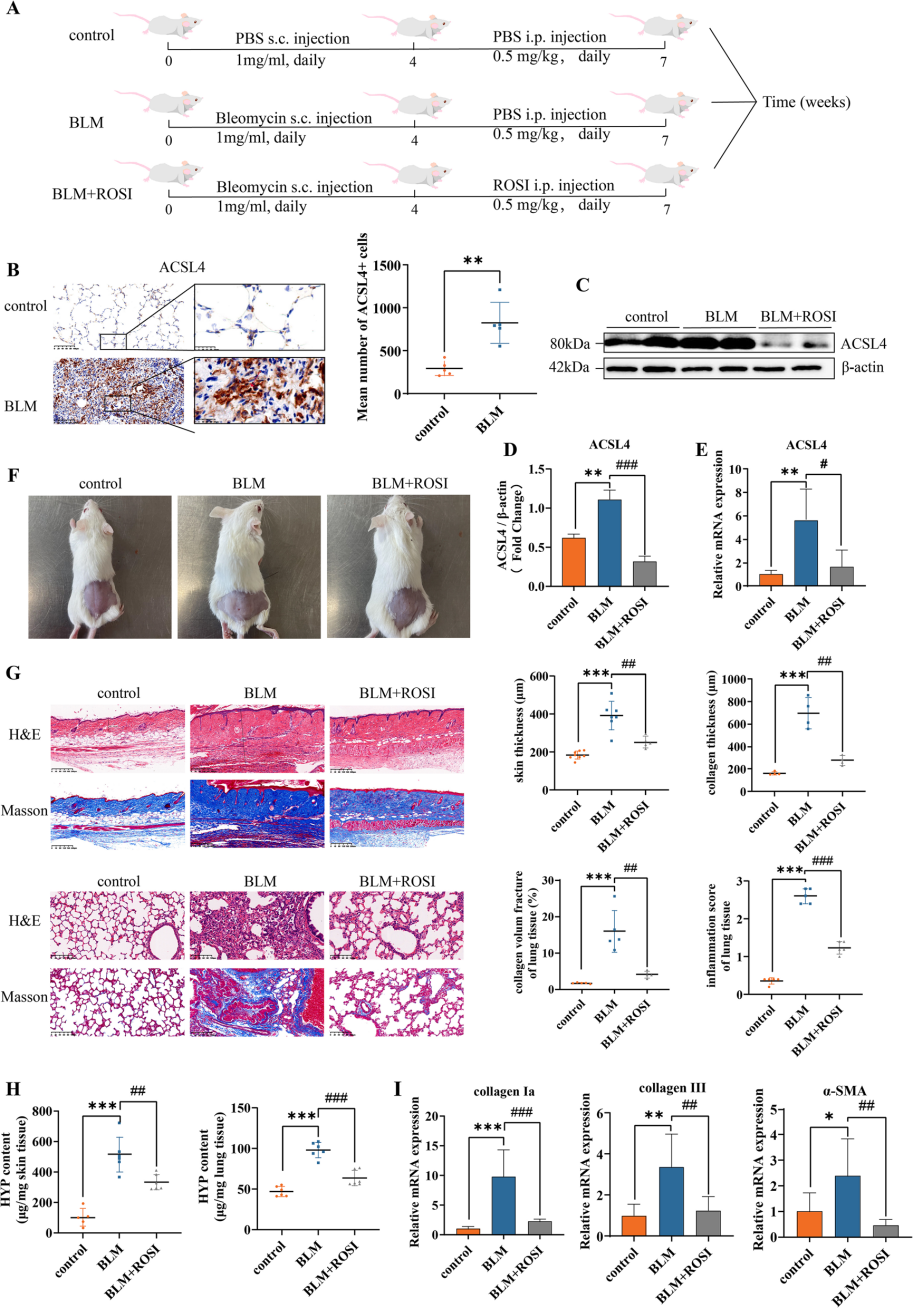
Inhibition of ferroptosis driver ACSL4 attenuates SSc fibrosis. **A** Schematic illustration of the treatment schedule.** B** Representative images of immunohistochemistry staining for ACSL4 of murine lung tissue (× 200, × 800). **C**, **D** Western blots of ACSL4 expression level in murine lung tissue. **E** Relative mRNA expression level of ACSL4 by quantitative PCR (*n* = 6). **F** Representative photos of the local injected murine skin of each group. **G** H&E and Masson’s staining representative images of murine skin and lung tissue, data are summarized in graphs (× 200), (*n* = 8). **H** HYP content of skin and lung tissue (*n* = 6). **I** Relative mRNA levels of fibrosis markers in each group by quantitative PCR (*n* = 6). (Data are presented as Mean ± SD *p < 0.05, **p < 0.01, ***p < 0.001, * stands for control versus BLM group; #*p* < 0.05, ##*p* < 0.01, ###*p* < 0.001, #stands for BLM group versus BLM + ROSI-treated group)

Based on above data, western blotting analyses of skin and pulmonary lesions showed increased ACSL4 levels (Fig. 1A, E). Again the higher ACSL4 accumulation in BLM mice fibrotic pulmonary interstitium rendered a potentially regulatory effect by biochemical analyses (Fig. 2B).

Rosiglitazone (ROSI), a classic peroxisome proliferator-activated receptor-γ agonist, has been used for ACSL4 inhibition [12, 27]. It has been reported to possess anti-fibrotic effect multi organs, such as liver and lungs, and the underlying mechanism involves PTEN, NF-kB, and TGF-β1 [28–30]. Thus, we chose ROSI as a pharmacological inhibitor for ACSL4 in mice. At the end day of 4 weeks BLM injection, we started the intraperitoneal ROSI treatment, in order to obtain a slower fibrotic progressing after the fully exposure of fibrotic inducing cause. We used the most frequently injected dosage (daily, 0.5mg/kg, i.p., for 21 days) in related literature [27, 29, 30] and the treated group displayed no visible signs of side effects of the drug (Fig. 2A). Also, the ROSI-treated mice exhibited remarkably less ACSL4 expression on both protein levels (Fig. 2C, D) and mRNA (Fig. 2E) in local skin.

Mice with daily administration of ROSI appeared less visible local skin thickness, which was a typical manifestation to identify fibrosis throughout the injection period (Fig. 2F). The pulmonary histological study showed that the 1.0 mg/ml BLM group indicated a more homogeneous and diffuse involvement of the alveolar interstitium, for almost all the pulmonary interstitium of the inferior lobe was involved, even some of the slices showed partial involvement of the upper lobe (Fig. 2G). Our observations are congruent with those in human samples, which are reported as the preserved normal pulmonary architecture with diffuse alveolar septa thickening, and pulmonary arterioles with marked luminal narrowing by circumferential intimal fibroplasia [2]. Histological analysis showed that ROSI treatment protected dermal fibrotic progressing by reducing excessive collagen deposition, re-obtaining subcutaneous fat. Compared to untreated mice, mice with ROSI treatment exhibited significantly less dermal thickness, collagen accumulation, and hydropxyproline (HYP) content (Fig. 2G, H). Furthermore, pulmonary tissue of treatment mice showed a tendency to much less collagen deposition, inflammation infiltration, and HYP value (Fig. 2G, H). In line with the promising results in local skin, pulmonary fibrosis alleviation showed its more efficient and long-lasting beneficial effect for inner organs. In alignment with histological data, mRNAs of collagen Ia, collagen III, and α-SMA were significantly reduced among treated mice skin (Fig. 2I).

These findings together supported the protective effect of ROSI in a regular dosage, which ameliorated skin severity and pulmonary fibrosis, in the BLM-induced fibrotic progressing course. This suggested inhibition of ferroptosis driver ACSL4 held promise for potential therapeutic relief in SSc fibrotic context.

### M1 upregulates ACSL4 and increases its sensitivity to ferroptosis in SSc

Tissue inflammatory response and fibrotic reaction are closely interlinked. Inflammatory macrophage (M1) activation, infiltration, and recruitment are regarded as a main trigger for promoting fibroblasts proliferation and collagen synthesis, as well as stimulating adaptive immunity and prolonging the unwanted SSc chronic inflammation [2–4, 14, 15]. In our previous research, flow cytometry analysis revealed that the percentage of M1 (marked F4/80+MHC II+) was significantly higher than the M2 (marked F4/80+CD206+) in bleomycin-treated mice [19].

**Fig. 3:**
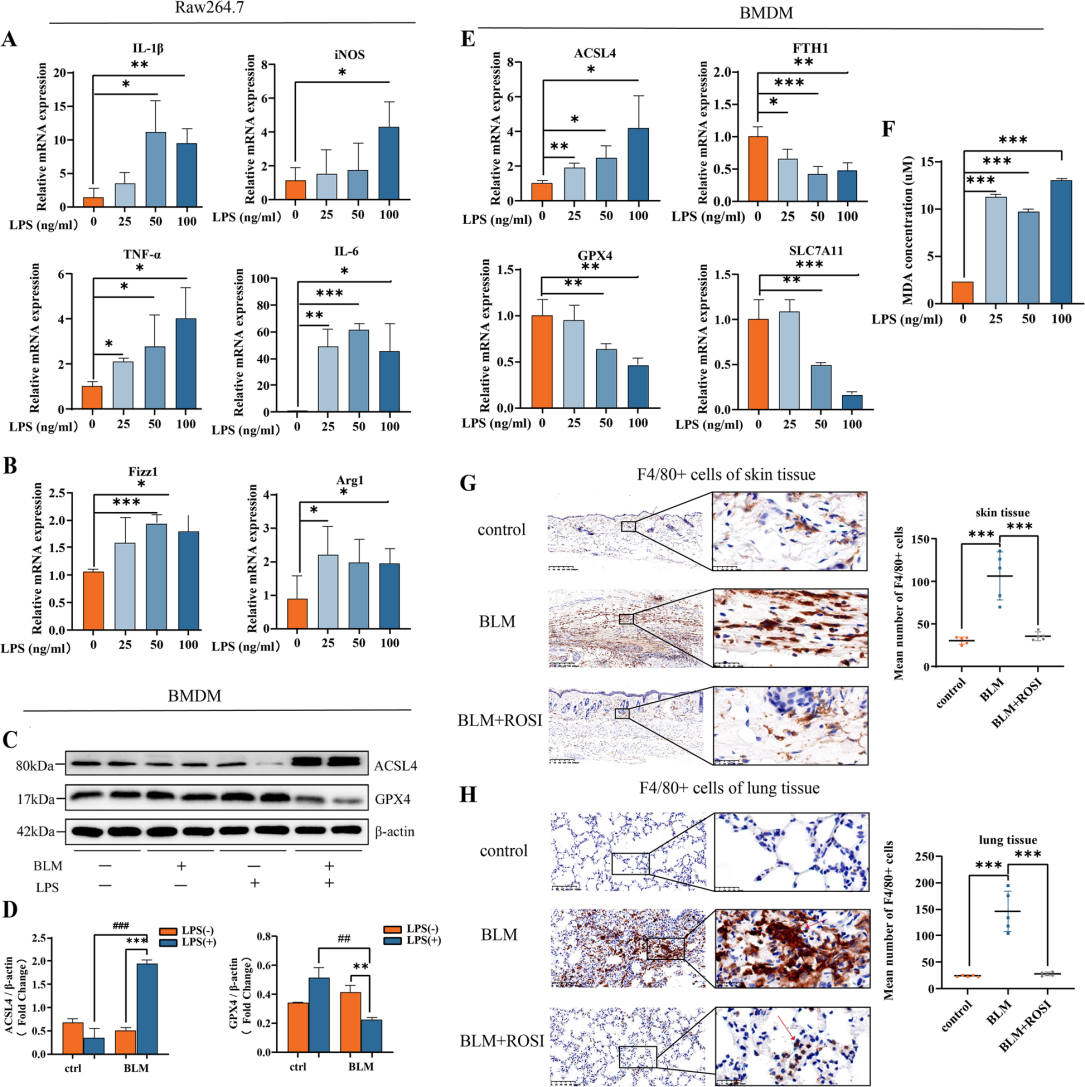
M1 upregulates ACSL4 and increases its sensitivity to ferroptosis in SSc. **A** Relative mRNA levels of M1 markers from Raw264.7 treated with different concentration of LPS. **B** Relative mRNA levels of M2 markers from Raw264.7 treated with different concentration of LPS. **C**, **D** Western blots of ferroptosis markers in BMDM derived from control or BLM group and then treated with PBS or LPS. **E** Relative mRNA levels of ferroptosis markers in BMDM treated with different concentration of LPS. **F** Relative MDA levels of BMDM treated with different concentration of LPS. **G**, **H** Representative images of immunohistochemistry staining for F4/80 of murine skin (× 100, × 800) and lung tissue (× 200, × 800) (*n* = 5). (Data are presented as Mean ± SD *p < 0.05, **p < 0.01, ***p < 0.001, * stands for control versus BLM group; #*p* < 0.05, ##*p* < 0.01, ###*p* < 0.001, #stands for PBS treatment versus LPS treatment)

To address the macrophage activation and polarization level in treatment of different LPS concentrations, we covered a range of LPS concentrations (0 ~ 100 ng/ml) for 24 h as indicated in literature to incubate Raw264.7 macrophage cell line [31–33]. Detection for major inflammatory cytokines, tumor necrosis factor alpha (TNF-α), interleukin 1 beta (IL-1β), interleukin 6 (IL-6), and inducible nitric oxide synthase (iNOS), revealed the significant overshooting inflammation response after LPS treatment (Fig. 3A). Meanwhile, to test whether proinflammatory macrophage (M1) transfered into tissue repair macrophage (M2) or not during increasing LPS incubation concentration, we have chosen inflammatory zone protein 1 (FIZZ1) and arginase 1 (Arg1) as putative markers of M2. However, different tendencies were shown as higher concentration of LPS, less M2 marker expression (Fig. 3B).

Since ACSL4 inhibition reduced tissue fibrosis in vivo (Fig. 2E–H), going in vitro, we decided to test the effect of ACSL4 on the ferroptosis of inflammatory macrophages (M1). First, we treated primary bone marrow-derived macrophages (BMDM) from healthy controls and BLM mice models with PBS or LPS stimulation (100 ng/ml). Subsequent western blotting of these BMDM revealed that only activated macrophages from BLM models showed ferroptosis tendency, with significantly increased expression of ACSL4 and decreased expression of GPX4, while the non-treated macrophages indicated no such response in protein levels (Fig. 3C, D).

Then, LPS in gradient concentration was used in BLM model-derived BMDM and following qPCR results showed that ferroptosis markers gradually changed with regard to different LPS concentration (Fig. 3E). And based on the above results in Raw264.7 cell line (Fig. 3A, B), the highest concentration of LPS, 100 ng/ml, induced most activated inflammatory macrophages (M1) and relatively less tissue repair macrophages (M2). Relatively, in 100 ng/ml group, BMDM showed the highest MDA level (Fig. 3F).

Last, according to the promising change fold of ACSL4 expression in BMDM, we explored whether its inhibition alleviated macrophage infiltration in vivo. Our data showed F4/80^+^ macrophage infiltration reduced in ROSI treatment group, both in skin (Fig. 3G) and pulmonary tissues (Fig. 3H).

### Calpain knockdown downregulates ACSL4 expression and rescues M1 ferroptosis

**Fig. 4:**
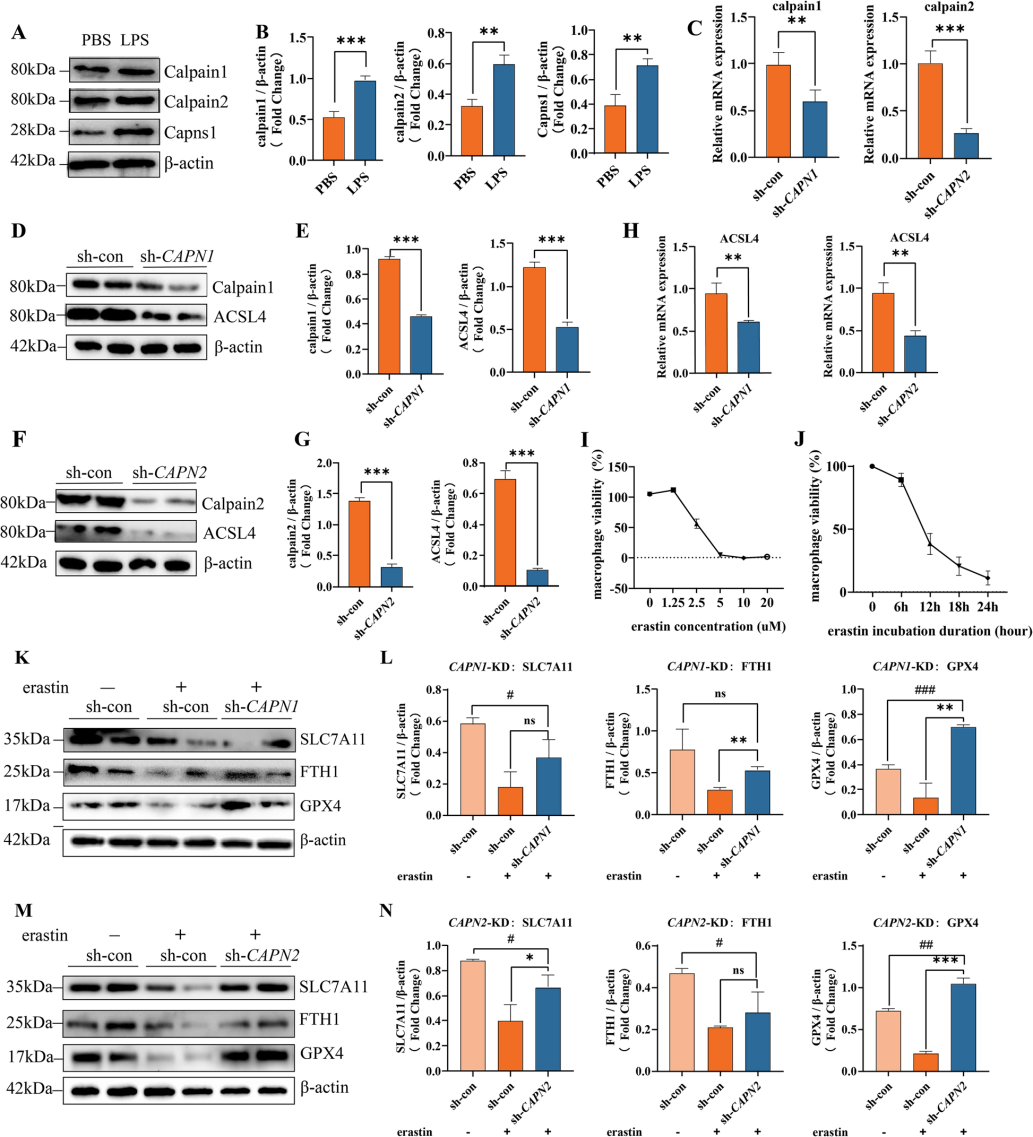
Calpain knockdown downregulates ACSL4 expression and rescues M1 ferroptosis. **A**, **B** Western blots of Calpain system in Raw264.7 treated with PBS or LPS. **C** Relative mRNA levels of Calpain 1 and Calpain 2 in Raw264.7 after *CAPN1* or *CAPN2* knockdown. **D**–**G** Western blots of Calpain 1, Calpain 2, and ACSL4 in Raw264.7 after *CAPN1* or *CAPN2* knockdown. **H** Relative mRNA levels of ACSL4 in Raw264.7 after *CAPN1* or *CAPN2* knockdown. **I**, **J** Raw264.7 cell viability after treated with erastin of different duration or concentration detected by CCK8. **K**–**N** Western blots of ferroptosis markers in Raw264.7 after *CAPN1* or *CAPN2* knockdown. (Data are presented as Mean ± SD *p < 0.05, **p < 0.01, ***p < 0.001)

The above in vitro studies revealed the importance of ACSL4 in macrophage ferroptosis and fibrosis. Among its massive potential regulators, calpain system was accidentally noticed as a candidate for its upstream activation in macrophage cell line Raw264.7 while we did related researches on calpains. Our previous research have proved increased serum calpain activity in SSc or SSc-ILD patients [22]. Western blotting and qPCR results revealed that the calpain 1, calpain 2, and their subunit capns1 were all upregulated in LPS-activated Raw264.7 cells (Fig. 4A–C). These background knowledge suggested a calpain activating situation in SSc.

The first interesting clue of calpain system’s the potential regulatory effect on ACSL4 was that when we knockdowned calpain 1 or calpain 2, LPS-activated Raw264.7 exhibited lower ACSL4 mRNA and protein levels vs. control group (Fig. 4D–H).

To test whether calpain knockdown can render a protective effect in ferroptosis-prone milieu, we evaluated ferroptosis levels of calpain knockdown macrophages when they faced a strong ferroptosis inducer, erastin. Before using erastin to establish an in vitro ferroptosis model for Raw264.7 cell line, we explored the suitable and safe drug concentration and duration for cell treatment. As literature suggested [34, 35], we tested a concentration range from 0 to 20 µM (Fig. 4I), as well as a duration range from 0 to 24 h (Fig. 4J) incubation using cell viability assay. The CCK8 results indicated a mature ferroptosis environment can be induced in 5µM for 24 h cellular incubation and this condition was established for the following detection.

Then, western blotting analyses results revealed a ferroptosis protective capacity of calpain knockdown, both calpain 1 or calpain 2, for providing a significant rescue effect on SLC7A11, FTH1, and GPX4 protein expression (Fig. 4K–N). Collectively, these results indicated that the calpain knockdown downregulated the increased ACSL4 and rescued macrophage ferroptosis in an erastin environment.

### ACSL4 inhibition abolishes overshooting calpain-mediated ferroptosis

**Fig. 5:**
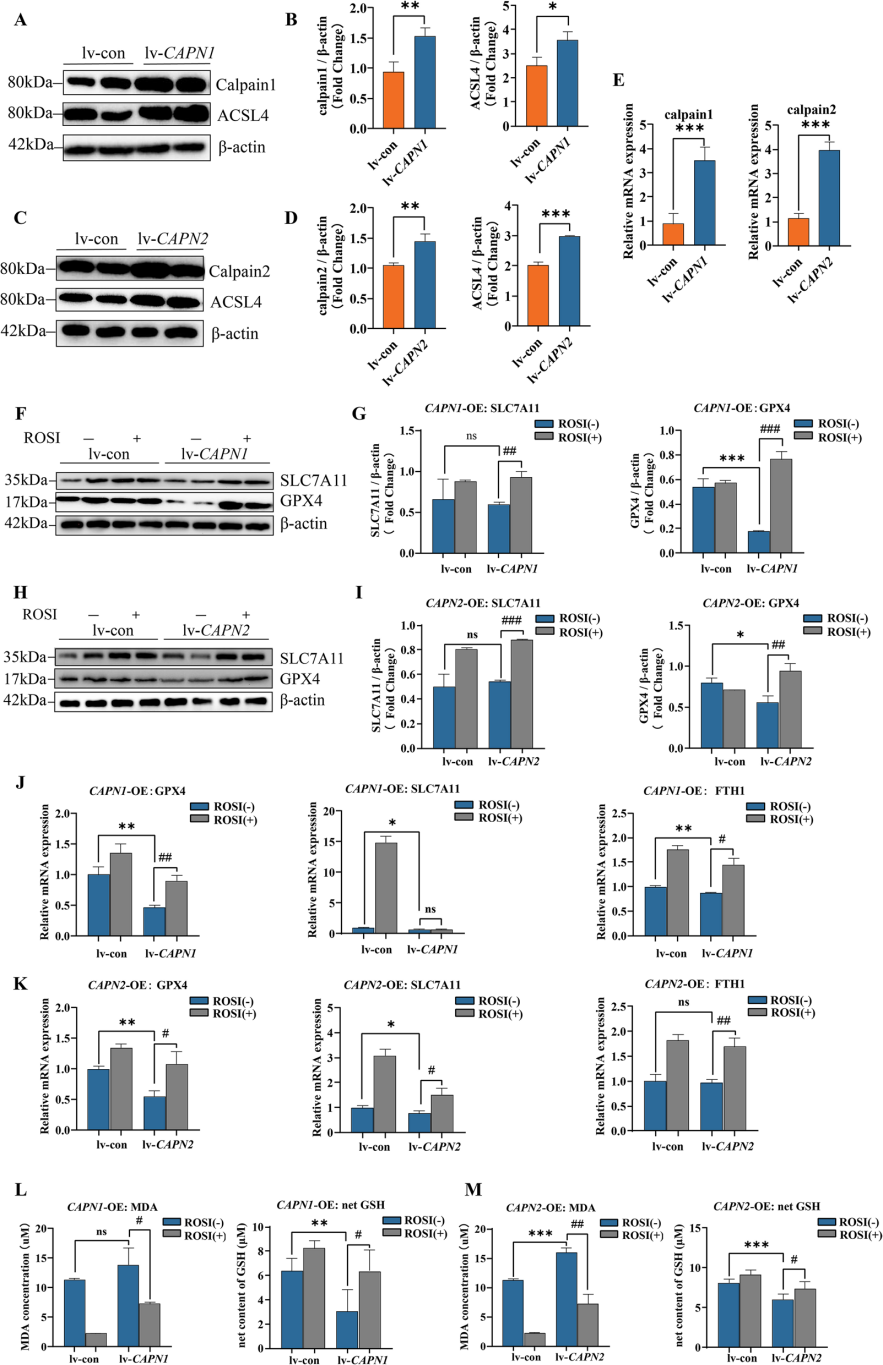
ACSL4 inhibition abolishes overshooting Calpain-mediated ferroptosis. **A**–**D** Western blots for Calpain system and ACSL4 in Raw264.7 after *CAPN1* or *CAPN2* over-expression. **E** Relative mRNA levels of calpain 1 and calpain 2 in Raw264.7 after *CAPN1* or *CAPN2* over-expression. **F**–**I** Western blots for ferroptosis markers in Raw264.7 after *CAPN1* or *CAPN2* over-expression with rescued by ROSI (1 μM, 24 h) or not. **J**, **K** Relative mRNA levels of ferroptosis markers in Raw264.7 after *CAPN1* or *CAPN2* over-expression with rescued by ROSI (1 μM, 24 h) or not. **L**, **M** Relative MDA levels and net GSH concentrations of Raw264.7 after *CAPN1* or *CAPN2* over-expression with rescued by ROSI (1 μM, 24 h) or not. (Data are presented as Mean ± SD *p < 0.05, **p < 0.01, ***p < 0.001, * stands for lv-control versus lv-over-expression group; #*p* < 0.05, ##*p* < 0.01, ###*p* < 0.001, #stands for control group versus ROSI incubated group)

To establish a calpain overshooting model in Raw264.7 cell line, we utilized lentivirus to over-express calpains, calpain 1 or calpain 2, and western blotting results revealed the expected tendency of increasing ACSL4 expression (Fig. 5A–E).

Then the well-established cells were subjected to ROSI treatment (1μM, 24h) to quest whether ACSL4 inhibition can rescue the overshooting calpain-induced ferroptosis tendency. After incubation, all the cell groups exposed to erastin medium (5μM, 24h) to induce ferroptosis. As the results showed, compared to over-expressing group, ferroptosis was significantly decreased in ROSI treatment. Incubation in ROSI remarkably rescued macrophage ferroptosis from protein and mRNA levels, MDA and GSH content (Fig. 5F–M).

These results together further indicated the regulatory relationship in calpain/ACSL4/ferroptosis axis, revealing an underlying interplay between calpain and ferroptosis signaling pathway.

### Calpain inhibition halts calpain-ACSL4 crosstalk and progressive fibrosis

**Fig. 6:**
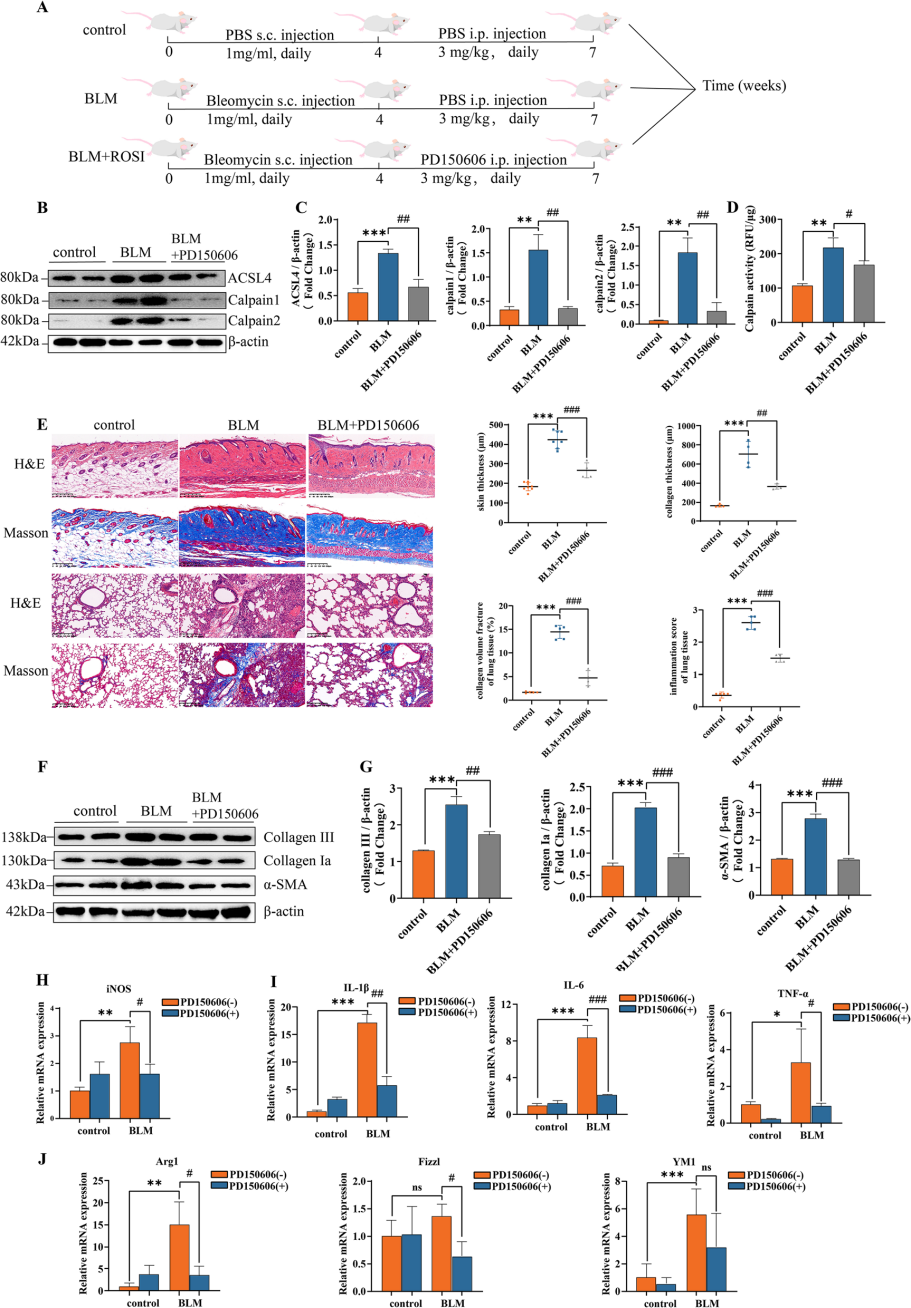
Calpain inhibition halts Calpain-ACSL4 crosstalk and progressive fibrosis. **A** Schematic illustration of the treatment schedule. **B**, **C** Western blots of calpain 1 and calpain 2 in murine skin tissue from each group. **D** Calpain activity in murine skin tissue from each group. **E** H&E and Masson’s staining representative images of murine skin and lung tissue, data are summarized in graphs (*n* = 8). **F**, **G** Western blots of fibrosis markers in murine skin tissue from each group. **H** Relative mRNA levels of ACSL4 from each group. **I**, **J** Relative mRNA levels of M1 and M2 markers from each group. (Data are presented as Mean ± SD *p < 0.05, **p < 0.01, ***p < 0.001, * stands for control versus BLM group; #*p* < 0.05, ##*p* < 0.01, ###*p* < 0.001, #stands for BLM group versus BLM + PD150606-treated group)

Since the interesting findings in Raw264.7 cell lines, we postulated that calpain inhibition would alleviate tissue ferroptosis thus providing a therapeutic effect in SSc mice model. We chose PD150606 as a pharmacological inhibitor for calpain as previously described [19, 36], and PD150606 was reported that correlated with PI3K/AKT1 signaling pathway in SSc BLM model [19]. To testify PD150606 as an effective inhibitor, we treated BLM-induced mice with PD150606 (daily, 3mg/kg, i.p., for 21 days) (Fig. 6A), and the inhibition effect was confirmed by Western blotting and calpain activity assay (Fig. 6B–D). In alignment with in vitro data, calpain inhibition with PD150606 effectively reduced ACSL4 protein level.

Then, the PD150606 treatment group effectively reduced BLM-induced fibrotic remodeling, as seen in histological results that less dermal thickness and collagen accumulation in skin and lungs (Fig. 6E). Protein analyses supported the histological observations. Again treatment with Calpain inhibitor associated with lower collagen Ia, collagen III, and α-SMA levels (Fig. 6F, G).

Going in vitro, qPCR results showed BMDM with PD150606 incubation significantly reduced ACSL4 expression (Fig. 6H). Also, after PD150606 incubation, both M1 and M2 cytokines were declined in BMDM, suggesting a reduced inflammation level (Fig. 6I, J), and there results were in line with our previous study [19].

In sum, systemic administration of calpain inhibitor PD150606 rendered protective effects for ACSL4 and ferroptosis, during the advancement of progressing fibrosis of SSc model. It ameliorated disease fibrotic severity through ACSL4 and ferroptosis. This holds promise for a potential target to relief progressing fibrosis in SSc.

## Discussion

In this study, we uncovered the following major findings: (1) Ferroptosis is present in the BLM-induced SSc mice model. (2) Inhibition of ferroptosis driver ACSL4 attenuates SSc fibrosis. (3) M1 upregulates ACSL4 and increases its sensitivity to ferroptosis in SSc. (4) Calpain knockdown downregulates ACSL4 expression and rescues M1 ferroptosis. (5) ACSL4 inhibition abolishes overshooting calpain-mediated ferroptosis. (6) Calpain inhibition halts calpain-ACSL4 crosstalk and progressive fibrosis. Continuing the previous findings in our team, we found a new potential intervention for fibrosis through ferroptosis in SSc models.

As our results have shown, macrophage infiltration was detected in the fibrotic tissues and after isolating macrophages in modeled mice, BMDMs were also detected cell ferroptosis sensitivity increase. Also, the content of cytokines secreted by macrophages was positively correlated with the degree of ferroptosis activity. This together supported macrophage ferroptosis is present and involved in the fibrosis advancing in SSc. However, recent bioinformatics research at the single-cell transcriptome level unveils high activity of ferroptosis in several cell types in SSc-ILD and SSc patients, especially in fibrobasts owing to abnormalities in aberrant extracellular matrix remodeling [5] and in immune cells modulating cell proliferation, differentiation, and migration [37]. Therefore, it can be inferred that the progression of fibrosis in SSc involves cell activation, death, and changes in ferroptosis activity of various cell types and it is hard to concluded into the effort of one single cell.

Apart from bioinformatics research, cell ferroptosis in the development and progression of SSc fibrosis has not been previously documented. So this study is a very new exploration to uncover the macrophage ferroptosis in the SSc fibrotic advancement and these results we present in this manuscript definitely lays foundation for the further investigation relating ferroptosis in other cell types in SSc and SSc-ILD.

There are few researches about BLM subcutaneously injected for the ferroptosis detection, while ferroptosis detection in BLM intratracheally injection induced pulmonary fibrosis model has been researched recently [38–41]. However, little literature reported the BLM subcutaneously injection approach can induce ferroptosis in local dermal and inner organ pulmonary tissue. Thus, in our study, we established two concentrations of 0.5mg/ml BLM and 1mg/ml BLM groups to mimic the moderate and severe disease condition. Although ferroptosis can be detected in tissues of both, ferroptosis degree presented by lipid peroxidation, redox potential content, iron levels are varying. 0.5 mg/ml BLM-injected group (moderate group) shows less significant ferroptosis sensitivity as less MDA accumulation and less GSH decline in skin and lung tissues. However, severe group indicates a more significant increasing ferroptosis level in tissue and this mice group is used for the rest of the detection. The inner link of this different tendency need to be further studied.

The mechanisms involved in SSc tissue fibrosis are not completely understood, and the effects of drugs on tissue fibrosis are unsatisfactory. Ferroptosis, as a novel programmed cell death, is implicated in various organ chronic fibrosis, like liver [42–44], lungs [38–41, 45], heart [46, 47], and renal [48], which shows multifaceted effect of promoting and inhibiting in these varying models and disease stages. In the BLM-induced pulmonary fibrosis models, a recent study indicate inhibition of ferroptosis by either liproxstatin-1 or deferoxamine (DFO) can alleviate the symptoms of pulmonary fibrosis [38]. Meanwhile, it also showed ferroptosis contributing to pulmonary epithelial damage in the early inflammatory phase of fibrosis [38], suggesting a successive involvement of fibrosis formation. In various pulmonary cell lines like alveolar type II cells [39], human fetal lung fibroblast 1 [40], and human bronchial epithelial cells [41], ferroptosis can be detected after modeling construction.

ACSL4 has been recently proved as a critical ferroptosis promoter in many non-tumor disease models, like organ intestinal/reperfusion [26, 49], ischemic stroke [50], acute kidney injury [51, 52], diabetic retinopathy [53], rhabdomyolysis [54], chronic prostatitis [55], and others. Moreover, its deficiency confers protection for inflammation [50, 51, 56] and immunity regulatory effect [57] in varying disease context. Notably, a recent research indicates its accumulation in psoriasis keratinocyte and keratinocyte ferroptosis induced by erastin show an inflammatory phenotype [56]. Meanwhile, ferroptosis has also been involved in the inflammation, chronic fibrosis, and mast cell activation in prostatitis [55]. These researches construct a bridge from inflammation to ferroptosis, which trigger our research of this phenotype in SSc context. Certainly, further studies into deeper pro-fibrotic mechanisms involved in ferroptosis and how these molecules link with each other are needed for illuminating this complex situation. Additionally, our in vitro and in vivo-based study findings need to be further verified in clinical tissue samples from SSc patients.

Taken together, ferroptosis intervention by ACSL4 inhibition or other underlying candidates in a preclinical setting represent a substantial step forward to the instructive and effective therapies for SSc and other fibrotic situation. Importantly, by revealing a fibrosis-independent phenotype, our data underscore mechanistic diversity and the complex interplay situation in inflammation-fibrosis feedback loop, which is already an active part of research among rheumatic diseases field. This study will open a new snap for fibrosis research in SSc.

## Conclusion

Collectively, our study provides evidence on the macrophage ferroptosis in animal model of systemic sclerosis. Our research focused on the ACSL4 upregulated in M1 ferroptosis and its effect on aggravating fibrosis progressing. Systematically injecting ROSI to inhibit ACSL4 showed effect on alleviating tissue fibrosis and abolishing overshooting calpain-mediated ferroptosis. Also, calpains may be the upregulator of ACSL4 over-expression, while calpain inhibition halted calpain-ACSL4 crosstalk and progressive fibrosis. Thus, here we offer insights that should be considered for the future researches on development of SSc fibrosis.

### Supplementary Information

**Additional file 1: Supplementary Table 1.** Sequences for lentivirus construction. **Supplementary Table 2.** Primer sequences for qPCR.

**Additional file 2.**

## Data Availability

The datasets generated and/or analyzed in this study are available from the corresponding author upon reasonable request.

## Abbreviations

SSc: Systemic sclerosis
BLM: Bleomycin
MDA: Malondialdehyde
GSH: Glutathione
ACSL4: Acyl-CoA synthetase long-chain family member 4
BMDM: Bone marrow-derived macrophages
LPS: Lipopolysaccharide
ILD: Interstitial lung disease
PCD: Programmed cell death
SLE: Systemic lupus erythematosus
PUFAs: Polyunsaturated fatty acids
Mφ: Macrophage
ALOX5: Kinase (ERK)-dependent 5-lipoxygenase
LTB4: Leukotriene B4
iNOS: Nitric oxide synthase
ROS: Reactive oxygen species
IL-1β: Interleukin 1 beta
IL-6: Interleukin 6
M-CSF: Macrophage colony-stimulating factor
ROSI: Rosiglitazone
H&E: Hematoxylin and eosin
HPF: High-power field
CVF: Collagen volume fracture
FBS: Fetal bovine serum
GFP: Green fluorescent protein
HYP: Hydroxyproline
GSSG: Oxidized glutathione
IHC: Immunohistochemical
FTH1: Ferritin heavy chain 1
SLC7A11: Solute carrier family 7a member 11
GPX4: Glutathione peroxidase 4
TNF-α: Tumor necrosis factor alpha
FIZZ1: Found in inflammatory zone protein 1
Arg1: Arginase 1
DFO: Deferoxamine
