# Supplementary material for: ACSL4 inhibition prevents macrophage ferroptosis and alleviates fibrosis in bleomycin-induced systemic sclerosis model
Source: Arthritis Res Ther. 2023 Oct 26;25:212. doi: 10.1186/s13075-023-03190-9 (PMC10601156; doi:10.1186/s13075-023-03190-9)
Supplement: Supplementary file 1 — Additional file 1: Supplementary Table 1. Sequences for lentivirus construction. Supplementary Table 2. Primer sequences for qPCR. [file 13075_2023_3190_MOESM1_ESM.zip › Supplementary Table 1. Sequences for lentivirus construction_ESM.docx]

**Supplementary Table 1. Sequences for lentivirus construction**

| Gene | Sequence (5’-3’) |
| --- | --- |
| M-CAPN1-sh | CCCATTGGTTTCCATGTCTTT |
| M-CAPN2-sh | GCGGTCAGATACCTTCATTAA |
| M-control-sh | TTCTCCGAACGTGTCACGT |
| CAPN1-overexpression | NM_007600.3 2142bp |
| CAPN2-overexpression | NM_009794.3 2103bp |
| Control-overexpression | Empty plasmid vector |
