# Supplementary material for: ACSL4 inhibition prevents macrophage ferroptosis and alleviates fibrosis in bleomycin-induced systemic sclerosis model
Source: Arthritis Res Ther. 2023 Oct 26;25:212. doi: 10.1186/s13075-023-03190-9 (PMC10601156; doi:10.1186/s13075-023-03190-9)
Supplement: Supplementary file 1 — Additional file 1: Supplementary Table 1. Sequences for lentivirus construction. Supplementary Table 2. Primer sequences for qPCR. [file 13075_2023_3190_MOESM1_ESM.zip › Supplementary Table 2. Primer sequences for qPCR_ESM.docx]

**Supplementary Table 2. Primer sequences for qPCR**

| Gene | Primer | Sequence (5’-3’) |
| --- | --- | --- |
| ACSL4 | Forward | CCTTTGGCTCATGTGCTGGAACT |
|  | Reverse | CAGCGGCCATAAGTGTGGGTTT |
| FTH1 | Forward | AGTTGTATGCCTCCTACG |
|  | Reverse | ATCACGGTCTGGTTTCTT |
| GPX4 | Forward | CCTCTGCTGCAAGAGCCTCCC |
|  | Reverse | CTTATCCAGGCAGACCATGTGC |
| SLC7A11 | Forward | TTGTTTTGCACCCTTTGAC |
|  | Reverse | AAAGCTGGGATGAACAGTGG |
| Collagen Ia | Forward | TAAGGGTCCCCAATGGTGAGA |
|  | Reverse | GGGTCCCTCGACTCCTACAT |
| Collagen III | Forward | CAGGACCTAAGGGCGAAGATG |
|  | Reverse | TCCGGGCATACCCCGTATC |
| MMP13 | Forward | TGATGGCACTGCTGACATCAT |
|  | Reverse | TGTAGCCTTTGGAACTGCTT |
| Calpain1 | Forward | TCAACCTTCGGGAAGTCAGC |
|  | Reverse | CATGTCATCCCCTGCCAACT |
| Calpain2 | Forward | GCTGCCCTGTCAACTTCATC |
|  | Reverse | TTTCCAGACGGACCAAACAC |
| IL-1β | Forward | GAAATGCCACCTTTTGACAGTG |
|  | Reverse | TGGATGCTCTCATCAGGACAG |
| IL-6 | Forward | CTGCAAGAGACTTCCATCCAG |
|  | Reverse | AGTGGTATAGACAGGTCTGTTGG |
| iNOS | Forward | GTTCTCAGCCCAACAATACAAGA |
|  | Reverse | GTGGACGGGTCGATGTCAC |
| TNF-α | Forward | CAGGCGGTGCCTATGTCTC |
|  | Reverse | CGATCACCCCGAAGTTCAGTAG |
| Fizz1 | Forward | CCAATCCAGCTAACTATCCCTCC |
|  | Reverse | CCAGTCAACGAGTAAGCACAG |
| Arg1 | Forward | CTCCAAGCCAAAGTCCTTAGAG |
|  | Reverse | CCACCTGCAAGACCATCGAC |
| YM1 | Forward | GGGCATACCTTTATCCTGAG |
|  | Reverse | CCACTGAAGTCATCCATGTC |
| β-actin | Forward | AGCCATGTACGTAGCCATCC |
|  | Reverse | GCTGTGGTGGTGAAGCTGTA |
