# Supplementary material for: ACSL4 inhibition prevents macrophage ferroptosis and alleviates fibrosis in bleomycin-induced systemic sclerosis model
Source: Arthritis Res Ther. 2023 Oct 26;25:212. doi: 10.1186/s13075-023-03190-9 (PMC10601156; doi:10.1186/s13075-023-03190-9)
Supplement: Supplementary file 2 — Additional file 2. [file 13075_2023_3190_MOESM2_ESM.doc]

Dear editor:

These are all the original blot images involved in our manuscript. Unfortunately, some of them were cropped during the jigsaw puzzle process, retaining only the cropped images in our original data files, while others were reserved to be left uncut.

In our future research, we must pay attention to such problems and develop the habit of retaining original blot images. Sorry for the problems we caused!

Looking forward to hearing from you soon!

Kind regards,

Dianyu Cao

Fig 1A-1 Original image of ACSL4 in skin tissue.

Fig 1A-2 Original image of SLC7A11 in skin tissue.

Fig 1A-3 Original image of FTH1 in skin tissue.

Fig 1A-4 Original image of GPX4 in skin tissue.

Fig 1A-5 Original image of β-actin in skin tissue.

Fig 1E-1 Original image of ACSL4 in lung tissue.

Fig 1E-2 Original image of SLC7A11 in lung tissue.

Fig 1E-3 Original image of FTH1 in lung tissue.

Fig 1E-4 Original image of GPX4 in lung tissue.

Fig 1E-5 Original image of β-actin in lung tissue.

Fig 2C-1 Original image of ACSL4.

Fig 2C-2 Original image of β-actin.

Fig 3C-1 Original image of ACSL4 in BMDM.

Fig 3C-2 Original image of GPX4 in BMDM.

Fig 3C-3 Original image of β-actin in BMDM.

Fig 4A-1 Original image of Calpain1 in Raw264.7.

Fig 4A-2 Original image of Calpain2 in Raw264.7.

Fig 4A-3 Original image of Capns1 in Raw264.7.

Fig 4A-4 Original image of β-actin in Raw264.7.

Fig 4D-1 Original image of Calpain1 in Raw264.7.

Fig 4D-2 Original image of ACSL4 in Raw264.7.

Fig 4D-3 Original image of β-actin in Raw264.7.

Fig 4F-1 Original image of Calpain2 in Raw264.7.

Fig 4F-2 Original image of ACSL4 in Raw264.7.

Fig 4K-1 Original image of SLC7A11 in Raw264.7.

Fig 4K-2 Original image of FTH1 in Raw264.7.

Fig 4K-3 Original image of GPX4 in Raw264.7.

Fig 4K-4 Original image of β-actin in Raw264.7.

Fig 4M-1 Original image of SLC7A11 in Raw264.7.

Fig 4M-2 Original image of FTH1 in Raw264.7.

Fig 4M-3 Original image of GPX4 in Raw264.7.

Fig 4M-4 Original image of β-actin in Raw264.7.

Fig 5A-1 Original image of Calpain1 in Raw264.7.

Fig 5A-2 Original image of ACSL4 in Raw264.7.

Fig 5A-3 Original image of β-actin in Raw264.7.

Fig 5C-1 Original image of Calpain2 in Raw264.7.

Fig 5C-2 Original image of ACSL4 in Raw264.7.

Fig 5C-3 Original image of β-actin in Raw264.7.

Fig 5F-1 Original image of SLC7A11 in Raw264.7.

Fig 5F-2 Original image of GPX4 in Raw264.7.

Fig 5F-3 Original image of β-actin in Raw264.7.

Fig 5H-1 Original image of SLC7A11 in Raw264.7.

Fig 5H-2 Original image of GPX4 in Raw264.7.

Fig 5H-3 Original image of β-actin in Raw264.7.

Fig 6B-1 Original image of ACSL4 in Raw264.7.

Fig 6B-2 Original image of Calpain1 in Raw264.7.

Fig 6B-3 Original image of Calpain2 in Raw264.7.

Fig 6B-4 Original image of β-actin in Raw264.7.

Fig 6F-1 Original image of Collagen Iα in Raw264.7.

Fig 6F-2 Original image of Collagen III in Raw264.7.

Fig 6F-3 Original image of α-SMA in Raw264.7.

Fig 6F-4 Original image of β-actin in Raw264.7.
